# Cortical morphology and illness insight in patients with schizophrenia

**DOI:** 10.1007/s00406-021-01328-x

**Published:** 2021-09-13

**Authors:** Marie-Luise Otte, Mike M. Schmitgen, Katharina M. Kubera, Nadine D. Wolf, Stefan Fritze, Lena S. Geiger, Heike Tost, Ulrich W. Seidl, Andreas Meyer-Lindenberg, Dusan Hirjak, Robert C. Wolf

**Affiliations:** 1 Department of General Psychiatry, Center for Psychosocial Medicine, Heidelberg University, Vosstrasse 4, 69115 Heidelberg, Germany; 2 Department of Psychiatry and Psychotherapy, Central Institute of Mental Health, Medical Faculty Mannheim, Heidelberg University, Mannheim, Germany; 3 Department of Psychiatry and Psychotherapy, SHG-Kliniken Saarbrücken, Saarbrücken, Germany

**Keywords:** Schizophrenia, MRI, Surface-based morphometry, Gyrification, Cortical thickness, Insight

## Abstract

Insight into illness in schizophrenia (SZ) patients has a major impact on treatment adherence and outcome. Previous studies have linked distinct deviations of brain structure to illness insight, specifically in frontoparietal and subcortical regions. Some of these abnormalities are thought to reflect aberrant cortical development. In this study, we used cross-sectional data to examine associations between illness insight and two cortical surface markers that are known to follow distinct neurodevelopmental trajectories, i.e. cortical gyrification (CG) and thickness (CT). CG and CT was investigated in SZ patients (*n* = 82) and healthy controls (HC, *n* = 48) using 3 T structural magnetic resonance imaging. Illness insight in SZ patients was measured using the OSSTI scale, an instrument that provides information on two distinct dimensions of illness insight, i.e. treatment adherence (OSSTI-A) and identification of disease-related symptoms (OSSTI-I). CT and CG were computed using the Computational Anatomy Toolbox (CAT12). Whole-brain and regions-of-interest (ROI)-based analyses were performed. SZ patients showed higher CG in anterior cingulate, superior frontal and temporal gyrus and reduced CG in insular and superior frontal cortex when compared to HC. SZ patients showed decreased CT in pre- and paracentral, occipital, cingulate, frontoparietal and temporal regions. Illness insight in SZ patients was significantly associated with both CG and CT in the left inferior parietal lobule (OSSTI-A) and the right precentral gyrus (CG/OSSTI-A, CT/OSSTI-I). The data support a multi-parametric neuronal model with both pre- and postnatal brain developmental factors having an impact on illness insight in patients with SZ.

**Supplementary Information:**

The online version contains supplementary material available at 10.1007/s00406-021-01328-x.

## Introduction

Poor illness insight is a common feature of schizophrenia (SZ) and is associated with unfavourable long-term outcomes. Poor illness insight has been related to poor treatment adherence, poor clinical outcome, increased negative and positive symptoms and adverse integration into the community. In contrast, well-preserved illness insight has been linked to better metacognitive and psychosocial function, fewer relapses, decreased number of hospitalizations, and higher quality of life [36]. Previous studies have shown that illness insight is not a stable feature of the disorder but fluctuates over time, as a function of the natural course of the disease or modulated by treatment [40]. Although a plethora of studies so far have investigated illness insight in SZ, this clinically important feature still lacks a consistent, generally accepted definition. In this study, illness insight was defined as the patients’ ability to understand SZ as a disease they are suffering from, to correctly attribute symptoms as disease-related, and to accept the need of treatment and related treatment adherence.

Despite its clinical relevance, little is known about the neurobiological underpinnings of illness insight in SZ. In the last 2 decades, we have witnessed an increased effort to identify neuronal correlates of the different levels of illness insight, with varying results. Previous magnetic resonance imaging (MRI) studies on illness insight used distinct methodological approaches such as voxel- or surface-based morphometry (VBM, SBM) and reported alterations in networks comprising frontal, parietal, temporal, basal ganglia and cerebellar regions, respectively [11, 13, 36]. For instance, Cooke et al. reported positive associations between illness insight and grey matter volume (GMV) in temporal cortex, right parietal gyrus and precuneus [13]. SBM studies examining relationships between illness insight and cortical thickness (CT) reported associations between poor awareness of illness and lower CT in the left middle frontal and inferior temporal gyri, while poor awareness of treatment need and efficacy were found to be related to lower CT in the left medial frontal gyrus, precuneus and temporal gyri [11]. Symptom misattribution was associated with widespread variations in CT in frontal, occipital, temporal and parietal regions [10]. Finally, higher self-reflectiveness was associated with thinner CT in the right occipital cortex [10]. Emami et al. showed lower CT in the right superior temporal gyrus, insula and parahippocampal gyrus in SZ patients with low insight [16]. Yet, other researchers were not able to identify any associations between illness insight and brain structure or function [5, 6]. As an example, Beland et al. could not find any significant relationships between CT and illness insight [6].

To date, however, it is unclear whether poor illness insight in SZ is differentially linked to cortical features of distinct evolutionary and genetic origin such as cortical gyrification (CG) and CT [25, 47]. To fill this gap, this MRI study examined the relationship between inter-individual variations of illness insight and CG and CT in SZ patients. On one side, CG is developed in the early stages of brain development, particularly during weeks 24 and 32 of gestation and undergoes changes until the early postnatal period [4, 19]. On the other side, CT undergoes dynamic development during the first 2 decades of life and is modulated by genetic as well as environmental factors (e.g., drug abuse, trauma, urban upbringing, etc.) [20, 22, 52, 62]. Therefore, analysing CG and CT separately makes it possible to differentiate between early cortical neurodevelopment and other ongoing neurodevelopmental processes during adolescence and young adulthood. Furthermore, the separate examination of cortical measures with temporally different neurodevelopmental origin will help to decipher the different contributions of CG and CT to illness insight in SZ. In this study, we used structural MRI and SBM to investigate relationships between cortical morphology and illness insight in patients with SZ. Illness insight was measured using the German version of the “Osnabrueck Scale of Therapeutic Attitudes and Identification of Psychological Problems in Schizophrenia” (OSSTI) [31, 59], an instrument that allows the assessment of distinct insight dimensions that are also related to treatment adherence and not solely into insight whether a disease is present or not. Assuming a multi-parametric neural model of illness insight in SZ influenced by the prenatal as well as postnatal neurodevelopment, we expected to find significant associations between OSSTI scores and both CG and CT. More precisely, we predicted a significant association between OSSTI scores and both CG and CT alterations in brain regions, especially prefrontal and inferior parietal regions that have been previously linked to self-perception, self-awareness [21, 46, 60], and “anosognosia” [38, 45, 57].

## Methods

### Participants

In this study, the original sample size consisted of 135 participants. MRI data quality procedures (see below) led to the exclusion of five participants (four SZ, one healthy participant). Eighty-two patients with SZ and 48 healthy controls (HC) were considered in within- and between-group analyses; detailed demographic and clinical data are shown in Table 1. SZ patients were consecutively recruited from the Department of Psychiatry and Psychotherapy at the Central Institute of Mental Health in Mannheim, Germany. Inclusion criteria consisted of a diagnosis of paranoid SZ according to ICD-10 (F20.0) with stable medication for at least 2 weeks, right-handedness, and age between 18 and 65 years. Exclusion criteria were a history of any substance dependency except for tobacco. HC were recruited through advertisements and screened for major psychiatric disorders before being included. Clinical evaluation included ascertainment of personal and family history and detailed physical and neurological examination. None of the HC had a lifetime history of neurological or medical illness, head injury, or substance abuse. All included study participants were right‐handed according to the Edinburgh Handedness Inventory [42]. All procedures contributing to this work complied with the ethical standards of the relevant national and institutional committees on human experimentation and with the Helsinki Declaration of 1975, as revised 2008. The local ethics committee (Medical Faculty at Heidelberg University, Germany) approved the study. Written informed consent was obtained from all participants following a complete description of the study.

**Table 1:** Demographics and clinical scores

	SZ (*n* = 82)	HC (*n* = 48)	*p* value
Age in years: mean (SD)	38.5 (10.9)	33.3 (11.2)	**0.003 + **
Gender: male (%)	40 (48.8)	20 (41.7)	0.547*
Education years: mean (SD)	12.9 (2.3)	14.5 (1.6)	**< 0.001 + **
Duration of illness in years: mean (SD)	10.1 (10.2)		
OLZe in mg: mean (SD)	18.0 (10.3)		
OSSTI-I: mean (SD)	12.7 (4.4)		
OSSTI-A: mean (SD)	20.1 (5.8)		
PANSS negative: mean (SD)	15.7 (7.1)		
PANSS positive: mean (SD)	15.6 (6.8)		
PANSS general: mean (SD)	33.4 (8.6)		
PANSS total: mean (SD)	64.6 (18.3)		
PANSS item G12: mean (SD)	1.60 (1.12)		

Significant results are in bold

Values presented as mean (standard deviation (SD)). Statistic refers to comparison between patients and HC

*OLZe* Olanzapine equivalents, *OSSTI* Osnabrueck Scale of Therapeutic Attitudes and Identification of Psychological Problems in Schizophrenia, *PANSS* Positive and Negative Syndrome Scale, *SD* standard deviation

*Chi-square-Test, + Mann–Whitney *U* test

Illness insight was measured with the OSSTI by Krupa in 2005 [31, 59]. We chose this scale because it comprises two dimensions of illness insight separately, adherence (OSSTI-A) and identification of disease-related symptoms (OSSTI-I). The advantage of this scale compared to other scales is the separate analysis of adherence (as one of two sub-scales) and identification of disease-related symptoms in SZ. Using this parameter, our study was able to analyse the influence of adherence as a clinically decisive parameter of illness insight, as non-adherence is associated with higher relapse risk and hospitalization [43]. The OSSTI is a self-rated instrument that was developed by Krupa [31, 59] based on the “Scale to Assess Unawareness of Mental Disorder” (SUMD) [1], Birchwood's Insight Scale (BIS) [7] and the “Self-Appraisal of Illness Questionnaire” (SAIQ) [37]. The original version comprised 24 items (6-point Likert scales), which were assigned to three sub-scales (1: identification of psychotic symptoms; 2: need for treatment and compliance; 3: consequences of mental illness). The first discriminatory test was performed in a sample of 26 patients with schizophrenia spectrum disorders. It resulted in the complete exclusion of scale 3 and a further exclusion of six items. Specifically, the OSSTI is a self-report instrument that comprises ten Likert-scale (six levels) items in two factors (OSSTI-A and OSSTI-I; scores by factor were used in this study). As an example of the items in these factors, statements like “I am healthy and have no mental complaints.” or “I tell my friends/my family/my acquaintances about my mental problems to prevent misunderstandings.” are used in the OSSTI. In a study comprising 85 schizophrenia patients, an acceptable Cronbach’s α for standardized items of 0.79 was detected [59]. Krupa reported consistencies of α1 = 0.67 (items 2, 4, 6, 8) and α2 = 0.77 (items 1, 3, 5, 7, 9, 10) for the final version. However, further psychometric analyses could not take place due to the sample size, validity evidence was not provided so far. The study by Waldorf on 85 SZ patients detected a Cronbach’s *α* for standardized items of 0.79, mean item intercorrelation of 0.28 and significant correlation with PANSS G12 item of *r* = 0.54/*p* < 0.001. Further psychometric assessment included the Positive and Negative Syndrome Scale (PANSS) [28].

### Structural neuroimaging data acquisition

Structural data were acquired using T1-weigthed three-dimensional (3D) magnetization-prepared rapid gradient-echo at the Central Institute of Mental Health, Mannheim, Germany on a 3.0 Tesla Magnetom Tim Trio MRI scanner (Siemens Medical Systems) with the following parameters: flip angle 7°, echo time (TE) = 3.93 ms; repetition time (TR) = 2530 ms; inversion time (TI) = 1100 ms; FOV = 256 mm; slice plane = axial; slice thickness: 1 mm; resolution = 1.0 × 1.0 × 1.0 mm; number of slices 176.

### Data analysis

For data analyses, the Statistical Parametric Mapping analysis package (SPM12 version 7771; www.fil.ion.ucl.ac.uk/spm/software/spm12/; last access: 27/11/2020) and the computational anatomy toolbox (CAT12 version vcat12.7; dbm.neuro.uni-jena.de/cat/; last access: 27/11/2020) implemented in SPM12 for surface-based morphometry (SBM; i.e. CG and CT) were used. To ensure high data quality, all original images were subject to visual inspection, and further data quality assurance was conducted by examining sample homogeneity of individual surfaces. Participants with 2 or more standard deviations in the Mahalanobis distance were partly excluded.

Gyrification analyses were based on the absolute mean curvature (AMC) approach as implemented in CAT12 [35], which has been used in previous MRI studies analysing SZ [41, 54]. A 23 mm Full Width at Half Maximum (FWHM) smoothing to the resampled surface data for CG was applied. CT was extracted using a projection-based distance measure [14]. The resampled surface data were smoothed using an 18 mm FWHM.

To investigate the relationship between CT and CG, respectively, multiple regression analyses were performed using whole-brain data and the OSSTI scale. The two OSSTI domains (OSSTI-A and OSSTI-I) were separately investigated. Higher scores in both sub-scales refer to a better illness insight. Each model included age, gender and olanzapine equivalents (OLZe) as covariates. Overall illness severity was accounted for by including PANSS total score as a further covariate. Inference was based on a peak-level threshold of *p* < 0.005 (uncorrected at the voxel/vertex level), in conjunction with an empirically determined extent-threshold *k* based on Random Field Theory (i.e., expected voxels or vertices or cluster per contrast) based on SPM resolution elements. Stereotaxic coordinates of significant between-group differences are reported from maxima within a given cluster according to the Montreal Neurological Institute (MNI) template. Following vertex values, distinct anatomical regions emerging from the between-group comparisons were labelled according to the DK40 atlas [15].

To investigate whether cortical morphology in SZ patients differs from HC in regions exhibiting significant associations with illness insight, the two-sample *t* test design implemented in CAT12 was performed comparing SZ and HC using whole-brain data separately for CT and CG adjusting for age and gender (as above uncorrected threshold *p* < 0.005, extent-threshold = *k*, see above). These whole-brain analyses were complemented by a region-of-interest (ROI) based approach, after extracting CG and CT according to the DK40 atlas from regions showing a significant relationship with OSSTI-I or OSSTI-A in the aforementioned regression analysis. ROI-analyses were conducted and displayed offline using the R software environment for statistical computing (version 4.0.3; https://www.r-project.org/; last access: 15/11/2020) [55]. ANCOVAs were performed for each region separately using age and gender as covariates; if the test assumptions were not met, robust ANCOVAs were performed using age as covariate, *p* values were adjusted for multiple comparisons using Bonferroni correction. An uncorrected threshold of *p* < 0.05 was applied. For significant adjusted *p* values, effect sizes were calculated.

## Results

### Demographics and clinical scores

The groups significantly differed in age (*p* = 0.003, Mann–Whitney *U* test) and education years (*p* < 0.001, Mann–Whitney *U* test). A significant gender difference was not found (*p* = 0.55, Chi-Square-test). For further clinical details, see Table 1. Both OSSTI sub-scales correlated negatively with PANSS item G12, lack of judgment and insight (OSSTI-I: *R* = − 0.35, *p* = 0.0015; OSSTI-A: *R* = 0.28, *p* = 0.012, Pearson's correlation).

### Associations between CG and illness insight

**Fig. 1:**
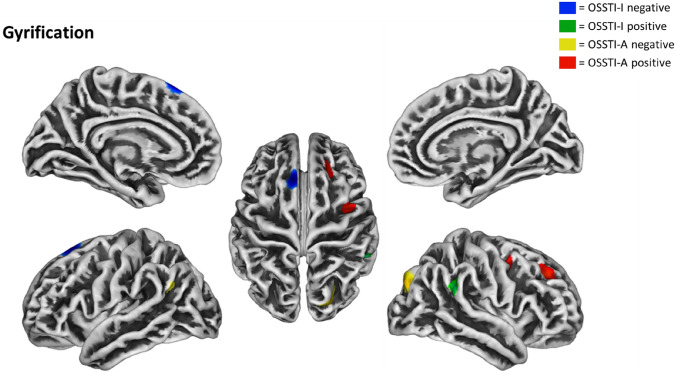
Negative and positive associations for CG and OSSTI-I and OSSTI-A. Results derived from whole-brain regression analyses implemented in SPM12, adjusted for age, gender, OLZe, PANSS (*p* < 0.005, uncorrected, expected voxels per cluster). *CG* cortical gyrification, *OLZe* Olanzapine equivalents, *OSSTI* Osnabrueck Scale of Therapeutic Attitudes and Identification of Psychological Problems in Schizophrenia (OSSTI-A adherence), *OSSTI-I* identification of disease-related symptoms), *PANSS* Positive and Negative Syndrome Scale

In SPM-based multiple regression analyses for OSSTI-A, significant negative association were found in the left inferior parietal lobule (IPL) and the right superior parietal gyrus, and significant positive associations were found in the right superior frontal and the precentral gyrus (M1). For OSSTI-I, significant negative associations were found in the left superior frontal gyrus and significant positive associations in the right supramarginal gyrus (see also Fig. 1 and Table 2).

**Table 2:** Associations between CG and OSSTI-A and -I in SZ patients

OSSTI	Directionality	Hemisphere	*p *value	*T* Score	Size (vertices)	Overlap (%)	Region
OSSTI-A	Negative	Left	0.00065	3.34	113	100	Inferior parietal
		Right	0.00081	3.27	132	100	Superior parietal
	Positive	Right	0.00114	3.16	139	69	Superior frontal
						31	Rostral middle frontal
			0.00155	3.06	88	52	Precentral
						48	Caudal middle frontal
OSSTI-I	Negative	Left	0.00024	3.66	88	100	Superior frontal
	Positive	Right	0.00082	3.27	123	67	Supramarginal
						33	Inferior parietal

Results derived from whole-brain regression analyses implemented in SPM12, adjusted for age, gender, OLZe, PANSS (*p* < 0.005, uncorrected, cluster-extent > k expected voxels per cluster). Anatomical labels follow the DK40 atlas

*DK40* Desikan–Killiany atlas, *OSSTI* Osnabrueck Scale of Therapeutic Attitudes and Identification of Psychological Problems in Schizophrenia (OSSTI-A adherence; OSSTI-I identification of disease-related symptoms)

### Associations between CT and illness insight

**Fig. 2:**
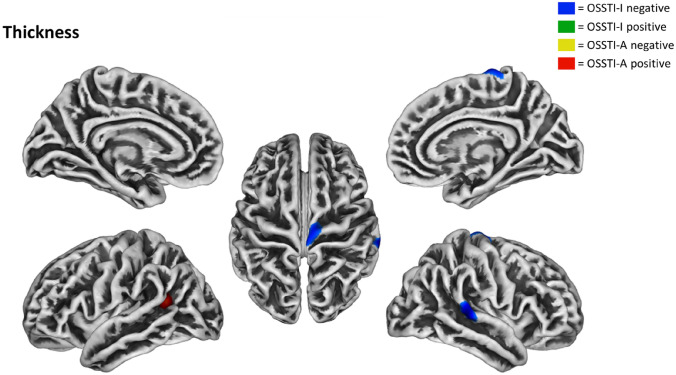
Negative and positive associations in the multiple regression analysis for CT and OSSTI-I and OSSTI-A. Results derived from whole-brain regression analyses implemented in SPM12, adjusted for age, gender, OLZe, PANSS (*p* < 0.005, uncorrected, expected voxels per cluster). *CT* cortical thickness, *OLZe* Olanzapine equivalents, *OSSTI* Osnabrueck Scale of Therapeutic Attitudes and Identification of Psychological Problems in Schizophrenia (OSSTI-A adherence; OSSTI-I identification of disease-related symptoms), *PANSS* Positive and Negative Syndrome Scale

Whole-brain regression analyses performed for OSSTI-A, revealed significant positive associations in the left IPL. For OSSTI-I, significant negative associations were found in the left pars triangularis of the inferior frontal gyrus and the right superior temporal gyrus and M1 (see also Fig. 2 and Table 3).

**Table 3:** Associations between CT and OSSTI-A and -I in SZ patients

OSSTI	Directionality	Hemisphere	*p* value	*T* score	Size (vertices)	Overlap (%)	Region
OSSTI-A	Positive	Left	0.00032	3.56	152	86	Inferior parietal
						14	Bankssts
OSSTI-I	Negative	Left	0.00187	2.99	91	41	Pars triangularis
						29	Insula
						24	Pars opercularis
						7	Lateral orbitofrontal
		Right	0.00027	3.61	182	55	Superior temporal
						45	Bankssts
			0.00123	3.13	122	66	Precentral
						34	Paracentral

Results derived from whole-brain regression analyses implemented in SPM12, adjusted for age, gender, OLZe, PANSS (*p* < 0.005, uncorrected, expected voxels per cluster). Anatomical labels follow the DK40 atlas

*DK40* Desikan–Killiany atlas, *OSSTI* Osnabrueck Scale of Therapeutic Attitudes and Identification of Psychological Problems in Schizophrenia (OSSTI-A adherence; OSSTI-I identification of disease-related symptoms)

### Comparisons with healthy controls

In comparison with HC regionally specific differences were found for both CG and CT (for more details, see supplements). In brief, SZ patients had higher CG in the frontal (left superior frontal gyrus) and the temporal lobe (left transverse temporal gyrus) and the cingulate (left rostral anterior cingulate) whereas lower CG was found in the frontal lobe (right superior frontal gyrus) as well as in both insulas compared to HC. For CT, SZ patients had significant lower CT in frontal (left precentral, paracentral, medial orbitofrontal gyrus and the right M1, rostral middle frontal and superior frontal gyrus), parietal (left superior parietal and right supramarginal gyrus), temporal (left inferior temporal gyrus) and occipital (left and right lateral occipital gyrus) regions as well as the cingulate (left posterior cingulate, right caudal anterior cingulate) in comparison with HC.

Finally, in ANCOVAs, there were significant differences for CT in the left IPL (*p* = 0.0262, Bonferroni corrected) and pars triangularis (*p* = 0.0463, Bonferroni corrected) as well as right superior temporal gyrus (*p* = 0.0441, Bonferroni corrected; see also supplementary material).

## Discussion

The main objective of this study was to examine associations between illness insight and CG and CT in SZ. We specifically focused on both symptom identification and treatment adherence. Two major findings emerged: first, we detected consistent associations between illness insight and both CG and CT in the left IPL and the right M1 only. Second, adherence and symptom identification dimensions showed distinct associations with brain morphology. In particular, the adherence domain showed significant associations with parietal (for CG: left inferior and right superior parietal gyrus (SPG); for CT left IPL) and frontal cortex (CG: right superior frontal gyrus and M1). Symptom identification was related to frontoparietal CG (right SPG, left superior frontal gyrus) as well as to CT in the parietal (right M1 and left pars triangularis) and the right superior temporal gyrus (STG).

Previous neuroimaging studies on the relationship between illness insight and brain morphology focused on GMV and CT. Although these studies used different analysis methods and yielded different results, they converged in brain regions such as frontoparietal, temporal, and cingulate cortex as well as precuneus [11] as crucial sites responsible for illness insight in SZ [13, 39, 49, 50]. Particularly noteworthy is the study by Beland et al., which did not find any correlations between CT and illness insight in SZ [6]. The authors suggested a limited role of CT and a greater role of psychological processes in the pathophysiology of poor illness insight in SZ [6]. Although the findings of abovementioned VBM studies might have been confounded by CT and cortical folding variations [32, 33, 58], thus limiting the comparability with the present study, we extend the findings of the previous studies by examining two cortical markers that show a distinct development over time, i.e. CG vs. CT.

Particularly noteworthy is the association between illness insight and CG and CT of the parietal cortex (e.g. involving IPL), suggesting both early neurodevelopmental variation as well as changes across adolescence and young adulthood. Two previous studies showed an association between illness insight and GMV and CT of the IPL [10, 13]. In particular, the IPL might play an essential role in the modulation of working memory, planning or problem solving [9, 51]. Aberrations in the non-dominant parietal gyrus additionally to frontal regions, are associated with anosognosia in numerous neurological diseases such as hemiplegia [38, 45]. The abovementioned functions may be linked to the functional coupling of the IPL within the so-called default mode network (DMN). DMN has been strongly implicated in self-reference and self-attribution [2], and its aberrant functioning has been frequently found in SZ patients [26]. CG and CT variations of the IPL highlight the relevance of this region for inter-individually varying illness insight, as much as they highlight possible similarities of illness insight in SZ and anosognosia in neurological diseases. Besides the IPL, the SPG was also found to be related to illness insight in this study. Both the IPL and SPG play an important role in executive cognition, particularly in working memory [12]. In addition, the supramarginal gyrus has been suggested to play a central role in processing information from different sensory modalities [29]. With its important role in different parts of working memory, variations in the parietal gyri might modulate sensory awareness and information processing along with subsequent decision-making, thus leading to various levels of recognition and self-attribution of a deviant health condition, i.e. SZ [23, 48] .

Furthermore, this study showed significant associations between OSSTI scores and CG in the right superior frontal, both CG and CT in M1 as well as CG in the left superior frontal gyrus and CT in pars triangularis. Since illness insight consists of at least three different dimensions, i.e., psychical, emotional, and somaesthetic [56], these findings support the neurobiological basis of this model for several reasons: first, the superior frontal region plays a crucial role in introspection, self-judgment, and self-awareness [21]. From a pathomechanistic perspective, aberrant frontoparietal structure in SZ might modulate the processes of self-reflexion/-awareness (recognition) and emotional capacities (feeling into oneself) leading to impaired illness insight [53]. For instance, Sapara et al. [49] showed that reduced GMV of superior, inferior and orbitofrontal regions are correlated with lower illness insight in SZ patients. A later study by Parellada et al. [44] corroborated these findings by showing that illness insight of early-onset first-episode psychosis patients depends on frontoparietal GMV. Interestingly, Buchy et al. [10] found misattribution of symptoms to be associated with CT in orbitofrontal cortex (OFC) and dorsolateral prefrontal cortex (DLPFC). Second, M1 variations might cause an impairment of somaesthetic body awareness apart from a less efficient control of sensorimotor functioning. Third, the pars triangularis of the inferior frontal gyrus (or ventrolateral prefrontal cortex) is responsible for two different forms of cognitive control, i.e. active retrieval and proactive interference resolution [30, 53]. Difficulties of active retrieval can have a negative impact on the process of self-reflection/awareness and lead to poor illness insight. Aberrant proactive interference resolution may contribute to misinterpretation of sensory information and difficulties to differentiate between an internal or external source of movement or sound as is known in patients with severe psychotic symptoms. These findings highlight the possibility of modulating illness insight via suppressing psychotic symptoms by antipsychotic medication or with specific psychotherapeutic interventions, which above all strengthen the cognitive control (e.g. metacognitive training) [40]. This said, decreasing the positive symptoms load and increasing the cognitive capacity (using cognitive training strategies) to attribute psychotic symptoms to an internal source may be helpful to increase illness insight and strengthen patients’ adherence. Finally, we found a negative association between CT in the right STG and OSSTI-I score. Few studies found associations between the ability to recognize experiences as abnormal [13] or direct associations between illness insight and GMV in STG [16, 39]. For instance, Buchy et al. [11] showed cortical thinning of temporal gyri in first-episode psychotic patients with insight deficits. Interestingly, fronto-temporal transcranial direct current stimulation (tDCS) optimizes attitudes toward mental illness [27]. Eventually, poor illness insight is not unique for SZ patients, but is also a well-recognized clinical phenomenon in patients with obsessive–compulsive (OCD) and eating disorders (ED). Few MRI studies explored the neurobiological origin of poor illness insight in OCD. According to Fan et al. [17], OCD patients with poor insight showed reduced amplitude of low-frequency fluctuation (ALFF) in left middle temporal gyrus and right STG, as well as increased ALFF in right middle occipital gyrus compared to OCD patients with good insight. Another fMRI study by Fan et al. [18] found decreased functional connectivity (FC) between anterior insula and medial OFC and increased FC between AI and dorsal anterior cingulate cortex (dACC) in OCD patients with poor insight compared to HC. More recently, Liu et al. [34] found that OCD patients with poor insight showed reduced CT in the left superior frontal gyrus, left ACC and right IPL, compared to HC. In ED, numerous studies focused on the neurocognitive basis of poor illness insight suggesting impaired self-awareness [3], cognitive-behavioural flexibility [61] and theory of mind [8]. Surprisingly, in sharp contrast to the data available in SZ or OCD, no MRI study so far specifically examined neural correlates of poor illness insight in ED. Given that poor illness insight is a transdiagnostic phenomenon, future studies should seek to expand extant models of illness insight well beyond SZ or OCD.

Strengths of this study include (i) the patient sample size, (ii) well-matched study groups, (iii) the use of advanced surfaced-based morphometry techniques for the investigation of cortical markers with different developmental trajectories. A potential limitation of this study is the cross-sectional study design, because illness insight is not a stable clinical feature, but depends on psychopathological symptoms and neurocognitive functioning and may fluctuate over time. Furthermore, given that CG is a rather stable neural trait, this marker may be indicative of vulnerability and risk to a higher degree than other structural features, e.g. grey matter volume or CT, that change over time as a function of age, environmental processes or disease-related factors. Nevertheless, in the light of this study’s findings, cortical surface feature of long-term temporal stability are linked to illness insight in stages of manifest disease, supporting the notion that CG abnormalities, if already present in vulnerable samples prior to manifest (first-episode) psychosis, could decisively modulate the degree of illness insight and related (long-term) clinical outcomes. This said, longitudinal studies investigating high-risk populations with both good and poor illness insight, heavy cannabis users prone to psychotic episodes and subsequent development of SZ, as well as first-degree relatives of SZ patients are needed to provide definitive conclusions.

## Conclusion

The results support a multi-parametric neuronal model of illness insight in SZ patients, suggesting an impact of working memory, introspection, self-awareness as well as sensory information processing. Especially, the IPL as part of the DMN might play an important role in the onset of inter-individually varying illness insight. As we found significant associations between both CG and CT, illness insight might be influenced by a neurobiological vulnerability, environmental influences in the brain development after birth as well as later structural brain changes, caused by the disease itself but also by other modulating factors such as antipsychotic medication [24]. This might provide opportunities to positively influence illness insight during the diseases progress—with antipsychotic medication or specific psychotherapeutic trainings.

## Supplementary Information

Below is the link to the electronic supplementary material.

Supplementary file1 (DOCX 36 KB)

## Data Availability

Data will be made available on scientifically reasonable request. Material will be made available on scientifically reasonable request.

## Abbreviations

ALFF: Amplitude of low-frequency fluctuation
AMC: Absolute mean curvature
BIS: Birchwood’s Insight Scale
CAT12: Computational Anatomy Toolbox
CT: Cortical thickness
CG: Cortical gyrification
dACC: Dorsal anterior cingulate cortex
DK40: Desikan–Killiany atlas
DLPFC: Dorsolateral prefrontal cortex
DMN: Default mode network
ED: Eating disorder
FC: Functional connectivity
FDR: False discovery rate
FOV: Field of view
FWHM: Full width at half maximum
GMV: Grey matter volume
HC: Healthy controls
IPL: Inferior parietal lobule
MNI: Montreal Neurological Institute
MRI: Magnetic resonance imaging
M1: Precentral gyrus
OCD: Obsessive-compulsive disorder
OFC: Orbitofrontal cortex
OLZe: Olanzapine equivalents
OSSTI: Osnabrueck Scale of Therapeutic Attitudes and Identification of Psychological Problems in Schizophrenia (originally in German: Osnabrücker Skala zu Therapieeinstellung und Identifikation psychischer Beschwerden bei Schizophrenie)
OSSTI-A: Adherence domain of OSSTI
OSSTI-I: Identification of disease-related symptoms domain of OSSTI
PANSS: Positive and Negative Syndrome Scale
ROI: Region of interest
SAIQ: “Self-Appraisal of Illness Questionnaire”
SBM: Surface-based morphometry
SPG: Superior parietal gyrus
SPM12: Statistical parametric mapping
STG: Superior temporal gyrus
SUMD: Scale to assess unawareness of mental disorder
SZ: Patients with schizophrenia
tDCS: Fronto-temporal transcranial direct current stimulation
TE: Echo time
TI: Inversion time
TR: Repetition time
VBM: Voxel-based morphometry

## Code availability

Data will be made available on scientifically reasonable request.
