# Supplementary material for: Cortical morphology and illness insight in patients with schizophrenia
Source: Eur Arch Psychiatry Clin Neurosci. 2021 Sep 13;272(6):985–95. doi: 10.1007/s00406-021-01328-x (PMC9388450; doi:10.1007/s00406-021-01328-x)
Supplement: Supplementary file 1 — Supplementary file1 (DOCX 36 KB) [file 406_2021_1328_MOESM1_ESM.docx]

*European Archives of Psychiatry and Clinical Neuroscience*

Supplementary material for

**Cortical morphology and illness insight in patients with schizophrenia**

Marie-Luise Otte^a^, Mike M. Schmitgen^a^, Katharina M. Kubera^a^, Nadine D. Wolf ^a^, Stefan Fritze^b^, Lena S. Geiger^c^, Heike Tost^c^, Ulrich W. Seidl^a, d^, Andreas Meyer-Lindenberg^b,c^, Dusan Hirjak^b,#^, Robert C. Wolf^a,*,#^

^a^  Department of General Psychiatry, Center for Psychosocial Medicine, Heidelberg University, Vosstrasse 4, 69115 Heidelberg, Germany

^b^ Department of Psychiatry and Psychotherapy, Central Institute of Mental Health, Medical Faculty Mannheim, Heidelberg University Mannheim, Germany

^c^ Department of Psychiatry and Psychotherapy, Research Group System Neuroscience in Psychiatry, Central Institute of Mental Health, Medical Faculty Mannheim, Heidelberg University, Germany

^d^ Department of Psychiatry and Psychotherapy, SHG-Kliniken Saarbrücken, Germany

^*^Corresponding author:

Robert Christian Wolf, MD

Center for Psychosocial Medicine

Department of General Psychiatry

Heidelberg University

Voßstraße 2, 69115 Heidelberg, Germany

E-mail: christian.wolf@med.uni-heidelberg.de

Tel: +49 - 6221/56-4405; Fax: +49-6221-564481

# D.H. and R.C.W. equally contributed to the manuscript.

**Supplementary Table 1a+b:** Group comparisons, SZ vs. HC (whole-brain) using two-sample t-test implemented in CAT12 adjusted for age and sex, performed separately for CG (a) and CT (b). Anatomical labels follow the DK40 atlas.

CAT12, Computational Anatomy Toolbox; CT, cortical thickness; CG cortical gyrification; DK40, Desikan–Killiany atlas; HC, healthy controls; SZ, patients with schizophrenia.

| **a. CG** |  |  |  |  |  |
| --- | --- | --- | --- | --- | --- |
|  |  |  |  |  |  |
|  |  | P-value | Size |  | Overlap of atlas region |
| SZ>HC | left | 0.00003 | 236 | 61.00% | **rostral anterior cingulate** |
|  |  |  |  | 34.00% | medial orbitofrontal |
|  |  |  |  | 3.00% | superior frontal |
|  |  | 0.00076 | 183 | 43.00% | **superior frontal** |
|  |  |  |  | 36.00% | caudal anterior cingulate |
|  |  |  |  | 21.00% | posterior cingulate |
|  |  | 0.00024 | 112 | 54.00% | **transverse temporal** |
|  |  |  |  | 46.00% | superior temporal |
| SZ<HC | left | 0.00007 | 304 | 71.00% | **insula** |
|  |  |  |  | 29.00% | superior temporal |
|  | right | 0.00187 | 110 | 100.00% | **superior frontal** |
|  |  | 0.0006 | 87 | 100.00% | **insula** |
|  |  |  |  |  |  |
| **b. CT** |  |  |  |  |  |
|  |  | P-value | Size |  | Overlap of atlas region |
| SZ>HC | left | 0 | 3409 | 26.00% | **precentral** |
|  |  |  |  | 24.00% | postcentral |
|  |  |  |  | 13.00% | pars opercularis |
|  |  |  |  | 11.00% | supramarginal |
|  |  |  |  | 6.00% | caudal middle frontal |
|  |  |  |  | 5.00% | insula |
|  |  |  |  | 5.00% | transverse temporal |
|  |  |  |  | 4.00% | rostral middle frontal |
|  |  |  |  | 3.00% | superior temporal |
|  |  |  |  | 2.00% | superior parietal |
|  |  | 0.00003 | 835 | 52.00% | **lateral occipital** |
|  |  |  |  | 27.00% | pericalcarine |
|  |  |  |  | 12.00% | cuneus |
|  |  |  |  | 6.00% | lingual |
|  |  |  |  | 3.00% | superior parietal |
|  |  | 0.00012 | 666 | 48.00% | **paracentral** |
|  |  |  |  | 28.00% | precentral |
|  |  |  |  | 23.00% | superior frontal |
|  |  |  |  | 2.00% | precuneus |
|  |  | 0.00006 | 158 | 97.00% | **posterior cingulate** |
|  |  |  |  | 3.00% | superior frontal |
|  |  | 0.00103 | 134 | 95.00% | **medial orbitofrontal** |
|  |  |  |  | 3.00% | superior frontal |
|  |  |  |  | 2.00% | rostral anterior cingulate |
|  |  | 0.00001 | 128 | 100.00% | **inferior temporal** |
|  |  | 0.00101 | 77 | 100.00% | **superior parietal** |
|  | right | 0.00001 | 4326 | 34.00% | **precentral** |
|  |  |  |  | 28.00% | postcentral |
|  |  |  |  | 13.00% | supramarginal |
|  |  |  |  | 10.00% | paracentral |
|  |  |  |  | 7.00% | pars opercularis |
|  |  |  |  | 7.00% | superior parietal |
|  |  |  |  | 2.00% | superior frontal |
|  |  | 0 | 1614 | 39.00% | **lateral occipital** |
|  |  |  |  | 16.00% | lingual |
|  |  |  |  | 12.00% | superior parietal |
|  |  |  |  | 11.00% | cuneus |
|  |  |  |  | 9.00% | pericalcarine |
|  |  |  |  | 7.00% | fusiform |
|  |  |  |  | 7.00% | inferior parietal |
|  |  | 0.00002 | 369 | 56.00% | **caudal anterior cingulate** |
|  |  |  |  | 40.00% | posterior cingulate |
|  |  |  |  | 4.00% | superior frontal |
|  |  | 0.00011 | 113 | 100.00% | **rostral middle frontal** |
|  |  | 0.00039 | 104 | 42.00% | **rostral middle frontal** |
|  |  |  |  | 29.00% | lateral orbito frontal |
|  |  |  |  | 29.00% | pars orbitalis |
|  |  | 0.00175 | 86 | 100.00% | **superior frontal** |
|  |  | 0.00077 | 82 | 100.00% | **supramarginal** |

Supplementary Table 2: ANCOVAs adjusted for age and sex (ROI-analyses). Robust ANCOVAs adjusted only for age

| thickness/gyrification | region | p-value unadjusted | p-value adjusted (Bonferroni correction) | effect size (partial eta squared) |
| --- | --- | --- | --- | --- |
| gyrification | left inferior parietal | 0.656 | 1 |  |
|  | left superior frontal | not significant* | not significant* |  |
|  | right supramarginal | 0.276 | 1 |  |
|  | right precentral | 0.159 | 1 |  |
|  | right superior parietal | not significant* | not significant* |  |
|  | right superior frontal | significant* | not significant* |  |
|  |  |  |  |  |
| thickness | **left inferior parietal** | **0.0026** | **0.0262** | **0.07** |
|  | **left pars triangularis** | **0.0046** | **0.0463** | **0.06** |
|  | **right superior temporal** | **0.0044** | **0.0441** | **0.06** |
|  | right precentral | significant* | not significant* |  |

*robust ANCOVAs
